# The smallest unit: effector and memory CD8^+^ T cell differentiation on the single cell level

**DOI:** 10.3389/fimmu.2013.00031

**Published:** 2013-02-15

**Authors:** Veit R. Buchholz, Patricia Gräf, Dirk H. Busch

**Affiliations:** ^1^Institute for Medical Microbiology, Immunology and Hygiene, Technische Universität MünchenMunich, Germany; ^2^Clinical Cooperation Groups “Antigen-specific Immunotherapy” and “Immune-Monitoring”, Helmholtz Center Munich (Neuherberg), Technische Universität MünchenMunich, Germany; ^3^Focus Group “Clinical Cell Processing and Purification”, Institute for Advanced Study, Technische Universität MünchenMunich, Germany; ^4^National Centre for Infection Research (DZIF)Munich, Germany

**Keywords:** T memory stem cell, single cell resolution, single cell fate mapping, subset diversification, memory ontogeny

## Abstract

CD8^+^ T cell immune responses provide immediate protection against primary infection and durable memory capable of rapidly fighting off re-infection. Immediate protection and lasting memory are implemented by phenotypically and functionally distinct T cell subsets. While it is now widely accepted that these diverge from a common source of naïve T cells (T_n_), the developmental relation and succession of effector and memory T cell subsets is still under intense debate. Recently, a distinct memory T cell subset has been suggested to possess stem cell-like features, sparking the hope to harness its capacity for self-renewal and diversification for successful therapy of chronic infections or malignant diseases. In this review we highlight current developmental models of memory generation, T cell subset diversification and T cell stemness. We discuss the importance of single cell monitoring techniques for adequately mapping these developmental processes and take a brief look at signaling components active in the putative stem cell-like memory T cell compartment.

## Introduction

In tissues with high cellular turnover, as for example in the epithelial layers of gut or skin, cells conveying the tissues major functional properties are constantly produced, mature and die (Creamer et al., 1961; Sun and Green, 1976; Celli et al., 2005). A general biological strategy for maintaining tissues subjected to such constant attrition is that of resupplying rapidly cycling short-lived cells from a source of long-lived, locally residing tissue stem cells (Simons and Clevers, 2011). Aside from their longevity, stem cells are characterized by the capacity to self-renew and in parallel generate a diverse offspring of short-lived cells for restocking the tissue's functional layers. Relatively short life spans of differentiated cells can also be observed for many branches of the hematopoietic system. Here, renewal processes can generally be tracked back to pluripotent bone marrow stem cells (Spangrude et al., 1988).

Naïve T cells (T_n_) also originate ultimately from hematopoietic stem cells. They are however, over time disconnected from their hematopoietic ancestors by thymic involution, which after puberty hinders further maturation of marrow-borne T cell precursors in the thymus (Steinmann, 1986; Hale et al., 2006). Further on, T cell receptor (TCR) recombination outfits developing T cells with unique epitope-specific receptors. This process of “individualization,” followed by some rounds of division, leaves a diverse TCR repertoire (Arstila et al., 1999; Casrouge et al., 2000). This repertoire is maintained, largely independent of hematopoietic precursors, by slow homeostatic turnover of naïve T cells (Jameson, 2002). While this steady state mode of homeostatic tissue maintenance lacks stem cell-like aspects of cell fate diversification, matters appear very different when naïve T cells are strained by infection or vaccination. Under these conditions, clonal T cell populations that recognize their cognate antigen expand vigorously and differentiate into various phenotypically and functionally distinct subsets (Williams and Bevan, 2007). This process can be described by analogy as the rapid outgrowth of an epitope-specific (mono- or oligoclonal) “tissue,” whose short-lived layers are quickly lost after resolution of infection, while its long-lived ones serve as a source for quicker and stronger responses to re-infection.

In this review we compile our current knowledge concerning the development and relation of acute and memory CD8^+^ T cell responses. A special focus is laid on the role of single cell fate mapping for adequately understanding these processes. In the first part we give a short overview on the diverse subsets present in the antigen-experienced T cell compartment and discuss how short-lived effector and long-lived memory T cells could arise from a limited number of epitope-specific naïve precursors. We continue by highlighting the importance of continuous observation and single cell resolution for an unambiguous evaluation of developmental pathways. In the last part we summarize current findings on stem cell-like signaling properties of distinct memory T cells and discuss further experimental routes for the evaluation of stemness in CD8^+^ memory T cells.

## Memory and effector subsets

Upon contact with their cognate antigen, naïve epitope-specific CD8^+^ T cells proliferate vigorously and differentiate to acquire phenotypic and functional properties that are key to resolving acute infection on the one hand and generating long lasting memory on the other hand. Initially it proved difficult to resolve whether these diverse properties are shared by all T cells responding to antigen challenge or are differentially assigned to distinct subsets (Dutton et al., 1998)—possibly even reserved to certain TCR specificities. The development of major histocompatibility complex (MHC) multimer technology (Altman et al., 1996) allowed for the first time to directly visualize endogenous antigen-specific CD8^+^ T cell responses irrespective of their functional status and revealed that T cell populations harboring TCRs specific to different epitopes of the same pathogen appear to expand, contract and enter the memory phase with similar kinetics (Busch et al., 1998). Recent data show that even very low affinity TCR peptide-MHC complex interactions suffice to generate memory CD8^+^ T cells, albeit after a weaker initial expansion (Zehn et al., 2009). Thus, largely independent of TCR specificity or affinity CD8^+^ T cell responses show a conserved pattern of expansion, contraction and memory maintenance.

**Figure 1:**
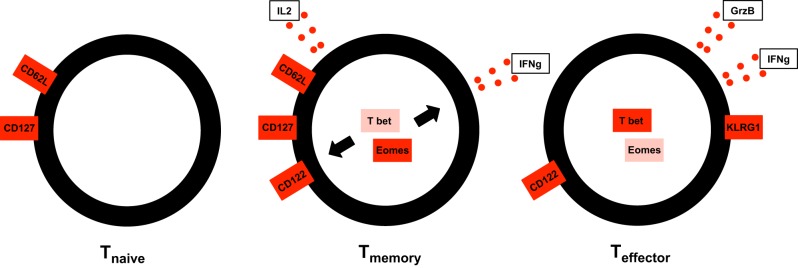
**Memory CD8+ T cells share traits of both the naïve and effector compartment.** Like T_n_, T_cm_ express lymph node homing molecule L-Selectin (CD62L) and IL7 receptor α chain (CD127) conveying recirculation capacity to secondary lymphoid organs and receptivity to homeostatic cytokine IL7. Like T_ef_, T_cm_ express IL15 receptor β-chain (CD122), and effector cytokine IFNγ. Both molecules are induced by transcription factors Tbet and/or Eomes.

It further became clear that already during the expansion phase endogenous (polyclonal) and adoptively transferred (monoclonal) TCR-transgenic T cell populations undergo a process of phenotypic und functional diversification that is indicative of their capability to transit into memory (Williams and Bevan, 2007). In general, memory T cells are characterized by their capacity to receive signals for homeostatic maintenance from the common gamma chain cytokines interleukin 7 (IL7) and IL15 (Schluns and Lefrançois, 2003). Like naïve T cells they express the IL7 receptor alpha chain (CD127) (Huster et al., 2004). They however are less dependent on homeostatic signals received from self-MHCI molecules than are naïve CD8^+^ T cells (Murali-Krishna et al., 1999) but more sensitive to IL15 through their expression of IL15 receptor alpha chain (CD122) (Schluns et al., 2002). While globally receptive to homeostatic cytokines IL7 and IL15, memory T cells can be further subdivided according to their capacity to migrate to secondary lymphoid organs and mount proliferative responses to re-infection (Sallusto et al., 1999). So-called “central memory T cells” (T_cm_) express lymph node homing molecules L-Selectin (CD62L) and chemokine receptor CCR7 and mount strong proliferation in response to re-infection. “Effector memory T cells” (T_em_) migrate to epithelial barriers and are capable of rapid effector function but only weak proliferation in response to antigen challenge (Masopust et al., 2001). By the time of peak primary expansion (usually around day 7 upon antigen challenge), the subdivision into CD62L^+^ CD127^+^ T_cm_ precursors, CD62L^−^ CD127^+^ T_em_ precursors and CD62L^−^ CD127^−^ effector T cells (T_ef_) that die during the contraction phase, is already apparent (Kaech et al., 2003; Huster et al., 2004). Even earlier, still during the expansion phase, the surface molecule killer cell lektin-like receptor G1 can be used to distinguish short- and long-lived T cells (Joshi et al., 2007). An important functional characteristic of memory precursor T cells is their capacity to produce interleukin 2 (IL2) (Sarkar et al., 2008). Initially IL2 signals during priming were described as prerequisite for successful CD8^+^ T cell recall expansion during memory (Williams et al., 2006). It was unclear however, whether the necessary IL2 was provided autocrine by CD8^+^ T cells themselves or in a paracrine manner by CD4^+^ helper T cells. Recently, it could be clarified that autocrine IL2 production during priming is a prerequisite for strong recall expansion of CD8^+^ T cells (Feau et al., 2011). This finding is well in line with the observation that IL2 production during the primary response serves as an indicator of vaccination success or protectivity in CD4^+^ (Darrah et al., 2007) and CD8^+^ T cell responses (Harari et al., 2004; Betts et al., 2006). In this context it is important to emphasize that IL2 producing CD8^+^ T cells are also capable of producing high amounts of effector cytokines like interferon γ (IFNγ) and tumor necrosis factor α (TNFα), creating a “per cell” pattern of cytokine production generally referred to as “multi-functionality” (Seder et al., 2008). While some aspects of the surface phenotype and cytokine receptivity of memory precursor T cells are clearly reminiscent of naïve T cells, transcriptional profiling (Holmes et al., 2005; Sarkar et al., 2008) and functional studies underline their developmental proximity to T_ef_. A possible aspect resolving this ambivalence was provided by research showing that master transcription factors T box transcription factor expressed in T cells (Tbet) (Szabo et al., 2000) and Eomesodermin (Eomes) (Pearce et al., 2003), which are essential for the induction of the effector cytokine IFNγ also induce expression of CD122, thus conveying IL15-receptivity to cells that have acquired effector properties (Intlekofer et al., 2005). Further research showed that while both transcription factors act redundantly in inducing effector function they appear to show reciprocal effects on the long-term maintenance of memory T cells. While increased expression of Tbet preferentially induces differentiation into short-lived effector T cells (Joshi et al., 2007), the presence of Eomes appears to support homeostatic memory maintenance (Banerjee et al., 2010; Zhou et al., 2010). Interestingly, during chronic infection with Lymphocytic choriomeningitis virus (LCMV), so called exhausted T cells, lacking the capacity for prolonged proliferation in response to antigen, show increased Eomes, and lower Tbet expression than their proliferation competent predecessors (Paley et al., 2012). This might hint to the fact that successful memory generation and maintenance are not exclusively dependent on one of these transcription factors, but rather on a specific balance of the two. Taken together, the data mentioned in this section point toward T cells having to acquire at least some effector characteristics during their ontogeny to achieve “T cell fitness” (Gett et al., 2003)—that is fitness to receive homeostatic maintenance signals and survive the contraction phase (see Figure 1). However, effector differentiation and strong proliferation can also be detrimental for transition to memory (Joshi and Kaech, 2008). Further on, the role of certain transcription factors for memory maintenance appears to differ greatly depending on the specific immunological context of chronic vs. cleared infection (Doering et al., 2012). Overall, the ambivalent positioning of memory CD8^+^ T cells in between effector and naïve states (Holmes et al., 2005) continues to yield controversy concerning their developmental path. This controversy focuses on two—currently unsolved—key conceptual questions: When during clonal expansion do long-lived memory and short-lived effector fates diverge? And, when are individual cells instructed to follow either one of these fates?

## The road to memory

**Figure 2:**
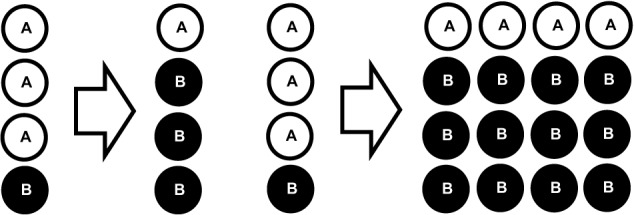
**Parallel proliferation hinders delineation of quantitative and qualitative changes in subset abundance. Left panel:** In the absence of proliferation or cell death, a change in subset phenotype from A to B can be inferred from the observation. **Right panel:** In the presence of proliferation (and/or cell death) this inference is not valid.

**Figure 3:**
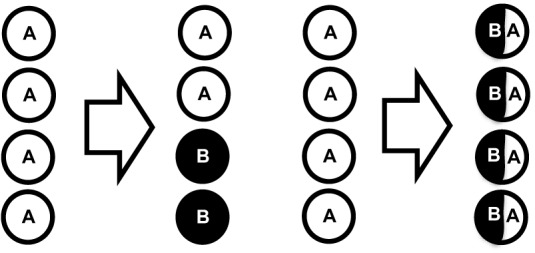
**Single cell resolution required to identify subset segregation. Left panel:** Global phenotypic composition changes (from 100% A to 50% A and 50% B) due to emergence of a subset expressing marker B instead of A. **Right panel:** Global phenotypic composition changes due to global change in expression of A and B.

**Figure 4:**
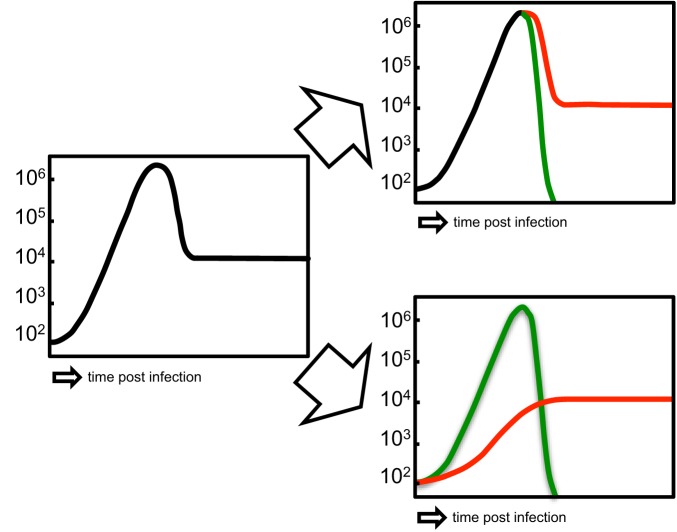
**Alternative proliferative histories of memory T cells. Left panel:** Overall primary expansion, contraction and memory maintenance of an epitope-specific T cell population. **Upper right panel:** The precursors of short-lived and long-lived subsets both contribute equally to primary expansion (“shared proliferative history”). **Lower right panel:** The precursors of long-lived subsets do not substantially contribute to primary expansion (“distinct proliferative history”).

In response to recognition of their cognate antigen, naïve CD8^+^ T cell populations expand and undergo phenotypic and functional diversification. Importantly, both processes occur in parallel, making it a difficult task to distinguish quantitative (cell proliferation or cell death) from qualitative changes (cell differentiation) as driving forces for distinct subset abundance. Thus, the dominance of phenotype A at an earlier time point of the immune response and that of phenotype B at a later one does not necessarily imply that cells of phenotype A have differentiated to B (see Figure 2). In order to elucidate the developmental path of a CD8^+^ T cell, the ultimate goal would be to monitor all the interaction, division and differentiation events that a single naïve T cell and its ancestors have experienced. While current approaches are still far away from achieving this total documentation of T cell history, some crucial insights have been gained using a variety of innovative technologies (Schumacher et al., 2010). Foremost, in order to discriminate global changes in population phenotype from the phenotypic segregation of subsets, technologies providing single cell resolution are warranted (see Figure 3). Flow cytometry or fluorescence microscopy both fulfill the prerequisite of single cell resolution and can routinely be used to investigate the expression of 10–15 different molecules per cell (Perfetto et al., 2004). The number of different markers that can simultaneously be detected by staining with fluorochrome-labeled antibodies is however, intrinsically limited due to the spectral overlap of excitation and emission spectra. This restriction has recently been considerably alleviated by a novel approach combining metal-labeled probes and mass spectrometry analysis. Here, heavy metal isotopes are used to label monoclonal antibodies and labeled cells are analyzed (with single cell resolution) for expression of 36 (theoretically up to 100) molecules by a combination of flow cytometry and mass spectrometry (Bendall et al., 2011). This so-called cytometry by time-of-flight approach provides a wealth of data that emphasizes the heterogeneous phenotypic and functional composition of epitope-specific T cell populations. While confirming previously defined CD8^+^ T cell subsets (T_n_, T_cm_, T_em_, and T_ef_) its major contribution to elucidating CD8^+^ T cell ontogeny is the definition of transitional states that lie in between major subsets and that connect T_n_ to T_cm_, T_cm_ to T_em_, and T_em_ to T_ef_ (Newell et al., 2012). While this observation is congruent with a progressive differentiation from naïve to memory to terminally differentiated T_ef_, it has to be emphasized that these data were gathered in subjects facing chronic and not acute infection. Thus, drawing conclusions concerning the developmental path from naïve to memory CD8^+^ T cells appears difficult. Further on, identifying transitional states as “missing links” situated on proposed developmental trajectories can only be a supplementary strategy to actually monitoring cells during this transition. Genetic approaches to “follow” CD8^+^ T cells of a certain phenotype or developmental state throughout their further developmental history, were pioneered by Baltimore and colleagues (Jacob and Baltimore, 1999). Recently, an even more stringently designed transgenic mouse model, linking the transient expression of effector molecule granzyme B (GrzB) to the permanent expression of enhanced yellow fluorescent protein (EYFP), has been developed (Bannard et al., 2009). While GrzB expression was found to be absent from epitope-specific memory CD8^+^ T cells at 7 weeks post infection with influenza virus, EYFP was readily detectable in a substantial fraction of the same cells. These memory T cells thus had passed through a state of effector functionality before reaching their GrzB^−^ memory state. According to these genetic single cell fate mapping data, memory T cells do not bypass effector differentiation completely. This is consistent with observations showing expression of GrzB by nearly all CD8^+^ T cells 2.5 days after acute LCMV infection (Sarkar et al., 2008), and with observations mentioned above that show a coupling of IL15 receptivity and effector cytokine IFNγ inducing transcription factors Tbet and Eomes. However, acquisition of effector function cannot be equated with induction of excessive proliferation and loss of longevity. Therefore, a key issue waiting to be resolved is to what end the precursors of memory take part in the massive proliferative expansion characteristic of an acute immune response (see Figure 4).

## The lone traveller

**Figure 5:**
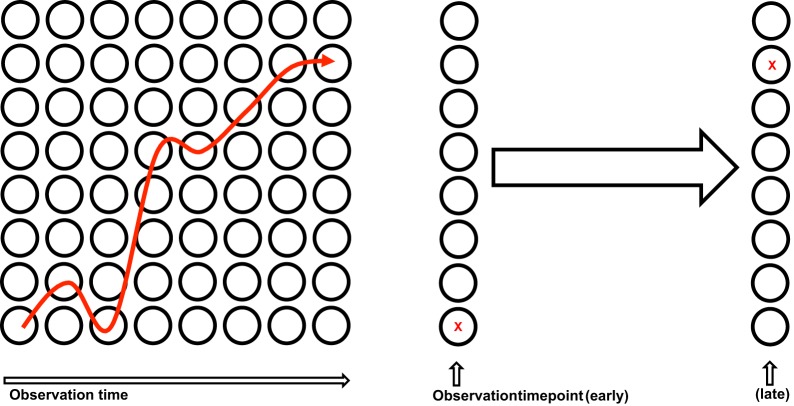
**Single cell fate mapping. Left panel:** Continuous monitoring of an individual cell (→). **Right panel:** Tagging of an individual cell by a heritable marker (X).

In order to adhere to the stem cell analogy introduced in the first paragraph of this review, naïve CD8^+^ T cells are not only required to generate diverse functional subsets and self-renew, but these capabilities should in principal converge within an individual precursor cell capable of generating a complete “epitope specific tissue.” For the hematopoietic system this capacity for tissue regeneration was shown in a pioneering study, in which the transfer of a single hematopoietic stem cell sufficed to repopulate diverse hematopoietic lineages after myeloablative treatment (Osawa et al., 1996). In order to investigate the developmental path of an individual T cell and its progeny under physiological conditions in immunocompetent hosts, two general approaches can be envisioned (see Figure 5): First, continuous observation and second, individualization by heritable markers (Stemberger et al., 2009; Buchholz et al., 2012). The obvious choice for continuously observing interaction, differentiation and proliferation of single cells and their daughters is intravital microscopy. Two-photon live imaging increased our knowledge concerning the initial events of single T cell development considerably (Henrickson and von Andrian, 2007). However, not every organ can be visualized equally well. Therefore, most insights gained by *in vivo* microscopy are currently based on studying immune reactions in lymph nodes draining the site of infection (Stoll et al., 2002). Here, quite representative tissue volumes can be analyzed. Three phases of T cell activation could be defined by this technique. “Phase 1” is characterized by transient contacts of antigen-specific T cells with their cognate peptide presented on MHC-complexes of dendritic cells (DCs). During this phase activation markers like CD44 and CD69 are already up-regulated by responding T cells. “Phase 2” is then marked by stable interactions in between T cells and DCs and coincides with the first production of cytokines. During “phase 3” transient contacts prevail again and T cells begin to divide (Mempel et al., 2004). It could be shown that increased peptide MHC complex density on DCs as well as increased numbers of peptide loaded DCs and higher peptide-TCR affinity shorten “phase 1” considerably and lead to a more rapid establishment of stable contacts (Henrickson et al., 2008). These data together with recent imaging studies implicate that after accumulating a certain amount of signal strength T cells are programmed for a defined developmental fate and then undergo proliferation (Beuneu et al., 2010; Moreau et al., 2012). This mode of signal integration (before proliferation) suggests a homogenous response of the progeny of a single T cell. A study applying multiple waves of antigen-presenting DCs could however show that further signal integration during the process of clonal expansion is possible (Celli et al., 2005). Another stem cell related mechanism of T cell diversification was first described by Reiner and colleagues. Here, the first cell division of *in vivo* activated T cells was imaged (Chang et al., 2007). Strikingly, it became apparent that T cell contacts with antigen presenting cells can lead to an asymmetric distribution of key components of the immunological synapse. After division this uneven distribution is thought to yield two daughter T cells that carry unequal amounts of defined signaling molecules and are fated to generate either short-lived effector (proximal daughter) or long-lived memory T cell progeny (distal daughter). This process has recently also been suggested to occur in memory T cells re-exposed to their cognate antigen (Ciocca et al., 2012) and is thought to be based at least in part on the asymmetric degradation of transcription factors due to the uneven concentration of the protein degradation machinery in one of the daughter cells (Chang et al., 2011). Moreover, asymmetric division was suggested to occur especially in the case of high affinity peptide TCR interaction, while low affinity interactions were biased for symmetric generation of “distal” memory fated daughters (King et al., 2012). These data implicate that a single T cell should be able to generate both effector and memory progeny and that the relative distribution of offspring onto these subsets is determined by the modes of division. However, formal proof for the importance of this partitioning mechanism for subset diversification and stem cell-like capacity of naïve and memory T cells is still lacking. It would require selective means of hindering asymmetric division while leaving other components of the immune response (e.g., peptide density, DC-T cell ratio, or peptide-TCR affinity) unchanged. A possible option to achieve this might be through interference with the orientation and positioning of the division plane as recently explored for the earliest divisions in embryonic development of *Caenorhabditis elegans* (Galli et al., 2011). Following the dynamic differentiation and proliferation process of single T cells via intravital microscopy is intrinsically limited by the volume of tissue monitored and the limited duration of observation. However, comprehensive single cell fate mapping beyond the earliest events of the immune response is possible by individualization. Two pioneering approaches have shed light on the diversification process of progeny originating from individual T cells during the expansion phase. The first approach truly visualizing the diversification potential that is inherent to a single naïve T cell used adoptive transfer of single naïve T cells outfitted with a heritable congenic marker to allow *in vivo* analysis of the diversification potential of individual T cells (Stemberger et al., 2007). Progeny generated from single precursor cells after infection with *Listeria monocytogenes* was here detected at the peak of clonal expansion by high sensitivity flow cytometry and analyzed for the expression of phenotypic markers (CD62L and CD127) and functional capacity (secretion of IFNγ, TNFα, and IL2). This study not only proved that it is technically feasible to identify single cell-derived progeny in a physiological animal model of acute infection, it also showed that the full diversity of effector and memory fates can originate from a single precursor cell. This obviously is at odds with the notion that T cell fate is determined during or even before the first cell division and rather hints to a continuous process of fate changing events acting upon the expanding progeny of a single naïve T cell. Importantly, a single cell-derived progeny can contain effector and memory subsets in parallel, thus adhering to the notion of stem cell-like capacity for diversification and self-renewal (Stemberger et al., 2009). Another study using the principal of heritably marking individual participants in an immune response is that of Schumacher and colleagues. The authors used an elegant method of integrating unique genetic tags (“barcodes”) into the genome of individual T cells and then measuring barcode abundance in progeny that develops during an *in vivo* immune response (Schepers et al., 2008). Drawing on a large library of unique barcodes (around 3000) this approach holds the obvious advantage of tracking hundreds of single cell-derived populations within one host. To date however, this approach is not capable of investigating phenotypic diversification within one single cell-derived progeny. When focusing on evaluating barcode abundance in progeny recovered from different organs at different times after acute infection, it was found that progeny originating from individual cells is capable of acquiring diverse homing patterns. Interestingly, effector and memory phase progenies gathered in the same experimental animals early and late after infection showed a similar barcode composition, which is again supportive of the idea that single naïve T cells have a stem cell-like potential for phenotypic, functional, and migratory diversification and can generate progeny both for the short and long-lived pool of antigen-experienced T cells (Gerlach et al., 2010). In the last part of this review, we will explore the similarities in between certain T cell subsets and stem cells by taking a closer look at shared traits on transcriptional and signaling level.

## Stem cell-like inside?

Compared to other antigen-experienced subsets, T_cm_ show enhanced longevity, self-renewal capacity, and proliferative potential as well as potency to generate T_ef_ and T_em_ cells. However, recent data suggest that only a small subset within the T_cm_ compartment is equipped with truly stem cell-like characteristics.

Several recent studies have been devoted on identifying parallels in between the subcellular organization of memory T cells and stem cells. The overexpression of multidrug efflux proteins of the ATP-binding cassette (ABC) superfamily in hematopoietic stem cells is—besides cell quiescence—one of the mechanisms mediating their resistance to cytotoxic drugs (Chaudhary and Roninson, 1991; Mizutani et al., 2008). An ABCB1-overexpressing CD161^+^ IL18Rβ^+^ cKit^+^ putative memory T cell subset was described in humans. This subset selectively survived chemotherapy and showed enhanced proliferative activity in a lymphopenic environment (Turtle et al., 2009). However, broader phenotypic characterization identified predominantly Vα7.2^+^ IL17-secreting mucosa-associated invariant T cells among the ABCB1 expressing T cells, arguing against the idea of CD161^+^ IL18Rβ^+^ cKit^+^ T cells being a less differentiated, stem cell-like reservoir of adaptive memory T cells (Dusseaux et al., 2011; Turtle et al., 2011; Havenith et al., 2012). Nonetheless, IL17-secreting subsets of CD4^+^ and CD8^+^ T cells appear to share some signaling pathways with stem cells: Signal transducer and activator of transcription 3 (STAT3) together with SMAD (human homolog of MAD “mothers against decapentaplegic” and SMA “small body size” protein) signaling mediates the polarization of CD4^+^ and CD8^+^ T cells toward IL17-secreting subsets and is also known to be active in stem cells. Interestingly, activation of STAT3 via IL21 suppresses terminal differentiation and exhaustion of T cells, highlighting its importance for sustained immune competence in the face of chronic infections (Leonard and Spolski, 2005; Li et al., 2005; Zeng et al., 2005; Fröhlich et al., 2009; Yi et al., 2009).

Further parallels in between memory T cells and tissue-specific stem cells were identified by a deeper investigation into shared transcriptional programs (Luckey et al., 2006; Fleming et al., 2008; Staal et al., 2008). In this context it was found that several molecular pathways found in stem cells, like Wnt/β-catenin, SMAD, STAT3, and forkhead box O (FOXO) signaling, are also active in T cells. These pathways appear to guide the generation of memory T cells through conserving their longevity, quiescence and self-renewal capacity (Betz and Müller, 1998; Castellino and Germain, 2007; Hand et al., 2010; Rao et al., 2010, 2012; Cui et al., 2011; Ji et al., 2011; Siegel et al., 2011; Yang et al., 2011; Kim et al., 2012; Thaventhiran et al., 2012).

The transcription factors lymphoid enhancer-binding factor 1 (LEF-1) and T cell factor-1 (TCF-1) are downstream targets of the Wnt pathway and are essential for a normal thymic maturation of naive T cells (Verbeek et al., 1995; Staal et al., 2008). The influence of active Wnt signaling on mature T cell differentiation came into focus as LEF-1 and TCF-1 are down-regulated upon T cell activation and are expressed in decreasing order in T_n_ →T_cm_→T_em_ subsets (Willinger et al., 2006; Gattinoni et al., 2009). Taken together proliferative activity, long-term survival and recall potential are affected by downstream targets of the Wnt/β catenin axis (Jeannet et al., 2010; Zhao et al., 2010; Zhou et al., 2010; Muralidharan et al., 2011; Xue and Zhao, 2012).

The enforced generation of less differentiated memory subset by the induction of Wnt signaling during priming and differentiation of naïve T cells (Zhang et al., 2005; Gattinoni et al., 2009) fueled further research into identifying such a stem cell-like undifferentiated subset under physiological conditions *in vivo*. Antigen-experienced cells with a surface phenotype characteristic for the naïve T cell compartment (CD45RA^+^ CD62L^+^ CCR7^+^ CD27^+^ CD28^+^ IL7Ra^+^) that in parallel express molecules associated with effector/memory T cell differentiation (IL2Rb, CXCR3, and CD95) were recently identified in human subjects (Gattinoni et al., 2011). Phenotypic and functional studies suggest enhanced self-renewal capacity and repopulation potential of these so called “T memory stem cells” (T_scm_) and propose them as an intermediate differentiation stage in between T_n_ and T_cm_ (Zhang et al., 2005; Gattinoni et al., 2011, 2009).

In normal homeostasis the pool of T_scm_ cells is believed to comprise a small fraction of 2–3% of all circulating T lymphocytes in human and non-human primates (Lugli et al., 2012). Their phenotype is especially enriched in antigen-specific T cell populations during the acute and chronic phases of immune responses as well as in resting memory (Gattinoni et al., 2011; Cieri et al., 2013). Furthermore, T_scm_ cells specifically accumulate in lymphopenic environments and during *in vitro* culture with a high availability of γ-chain cytokines IL2, IL7, IL15, and IL21 that control the homeostatic turnover of memory T cells (Zhang et al., 2005; Cieri et al., 2013). These observations suggest that instructive and permissive environmental signals provided by growth factors and cytokines, perhaps at site-specific niches, limit the size, and the stem cell-like potential of the memory T cell pool (Jiang et al., 2005; Li et al., 2005; Zeng et al., 2005; Mazzucchelli and Durum, 2007; Morrison and Spradling, 2008).

Possibly these stem cell-like memory T cells could provide a basic principle behind long-term memory maintenance, acting as a tiny reservoir of quiescent, long-lived cells that compensates for the continuous loss of more differentiated effector and memory T cells. However, this principle has never been experimentally proven. Strong T cell activation due to repetitive antigen encounter or chronic inflammation is usually associated with enhanced differentiation, exhaustion and induction of senescence (Wherry et al., 2003; Gattinoni et al., 2005; Appay et al., 2008; Sallusto et al., 2010; Wirth et al., 2010). Thus, a preservation of the regenerative potential within antigen-specific T cell populations by self-renewing, quiescent T_scm_ cells appears exceedingly important for maintaining immune responses against recurrent and chronic infections as well as malignancies. Providing optimally self-renewing T cells by distinct vaccination strategies or selective *in vitro* expansion of T cells out of patient material (Lugli et al., 2012), appears to open new avenues for immunological cell therapy approaches. Especially therapy of malignant diseases could be supported substantially by a self-renewing cellular source providing continuous anti-tumor activity (Gattinoni et al., 2012).

However, until today, only dynamics and properties of populations derived from the designated T_scm_ phenotype have been studied. The behavior and attributes of individual T_scm_ cells remain elusive and no conclusion can be drawn whether self-renewal capacity and multipotency are truly conjoint on the single cell level. A microscopic approach of continuous observation and single cell tracking in long-term cell culture, has proven very successful analyzing lineage development in the hematopoietic system; it could however, be difficult to adapt to the complex nature of *in vivo* T cell responses (Eilken et al., 2009). Adoptive *in vivo* transfer of single cells has already been successfully used to demonstrate that long-term reconstitution of the lymphohematopoietic system can be achieved by single hematopoietic stem cells (Osawa et al., 1996). Similarly, adoptive transfers of single naïve CD8^+^ T cells proved the multipotent differentiation potential of individual naïve T cells (Stemberger et al., 2007). A similar strategy could be implemented to study self-renewal and multipotency of memory T cells on the single cell level. Challenging the T_scm_ paradigm on the single cell level could contribute substantially to revealing how and to what extent stem cell-like regenerative potential is feeding the pool of memory T cells throughout life.

## Conclusion

New technologies allowing single cell *in vivo* fate mapping have started to provide novel insights into the diversification process from few naïve antigen-specific T cells into a spectrum of long- and short-living effector and memory T cell subsets. These studies suggest that subset diversity can even be generated from single naïve precursor cells and that this process provides in parallel to highly differentiated effectors a subset of T cells, which seems to resemble the differentiation plasticity of naïve T cells, so-called memory stem cell (T_scm_). However, physiological T cell responses are believed to be composed by the recruitment of multiple naïve precursor T cells. It is currently unknown whether under these conditions all recruited T cells follow the same diversification pattern or whether they differ on the single cell level. In addition, although the concept of the presence of a unique memory T cell subset with stem cell-like characteristics is intriguing, it still remains elusive to experimentally demonstrate that self-renewal capacity and multipotency can truly be conjoint on the single cell level. Although future studies are still necessary to further prove the novel concept of T cell subset diversification as well as memory T cell generation and maintenance, it is already obvious that these insights will have important implications for the improvement of active (vaccination) and passive (adoptive T cell transfer) immunotherapies for the treatment of infections, defined cancers and autoimmune disease.

### Conflict of interest statement

The authors declare that the research was conducted in the absence of any commercial or financial relationships that could be construed as a potential conflict of interest.
